# Deactivation of the inferior colliculus by cooling demonstrates intercollicular modulation of neuronal activity

**DOI:** 10.3389/fncir.2012.00100

**Published:** 2012-12-14

**Authors:** Llwyd D. Orton, Paul W. F. Poon, Adrian Rees

**Affiliations:** ^1^Institute of Neuroscience, Faculty of Medical Sciences, Newcastle UniversityNewcastle upon Tyne, UK; ^2^Department of Physiology, National Cheng Kung UniversityTainan, Taiwan

**Keywords:** inferior colliculus, cooling inactivation, auditory pathways, single unit, commissure, guinea pig, auditory processing

## Abstract

The auditory pathways coursing through the brainstem are organized bilaterally in mirror image about the midline and at several levels the two sides are interconnected. One of the most prominent points of interconnection is the commissure of the inferior colliculus (CoIC). Anatomical studies have revealed that these fibers make reciprocal connections which follow the tonotopic organization of the inferior colliculus (IC), and that the commissure contains both excitatory and, albeit fewer, inhibitory fibers. The role of these connections in sound processing is largely unknown. Here we describe a method to address this question in the anaesthetized guinea pig. We used a cryoloop placed on one IC to produce reversible deactivation while recording electrophysiological responses to sounds in both ICs. We recorded single units, multi-unit clusters and local field potentials (LFPs) before, during and after cooling. The degree and spread of cooling was measured with a thermocouple placed in the IC and other auditory structures. Cooling sufficient to eliminate firing was restricted to the IC contacted by the cryoloop. The temperature of other auditory brainstem structures, including the contralateral IC and the cochlea were minimally affected. Cooling below 20°C reduced or eliminated the firing of action potentials in frequency laminae at depths corresponding to characteristic frequencies up to ~8 kHz. Modulation of neural activity also occurred in the un-cooled IC with changes in single unit firing and LFPs. Components of LFPs signaling lemniscal afferent input to the IC showed little change in amplitude or latency with cooling, whereas the later components, which likely reflect inter- and intra-collicular processing, showed marked changes in form and amplitude. We conclude that the cryoloop is an effective method of selectively deactivating one IC in guinea pig, and demonstrate that auditory processing in the IC is strongly influenced by the other.

## Introduction

Sub-thalamic auditory processing in mammals is mediated by bilaterally organized pathways that originate with the entry of the auditory nerves into the cochlear nuclei and culminate in the inferior colliculi. On each side of the midline, the connections between multiple processing centers in the brainstem give rise to several parallel processing streams that converge in the inferior colliculus (IC) (for review see: Oliver and Huerta, 1992; Malmierca and Hackett, 2010). Although inputs from both left and right ears contribute to the pathway on each side, the representation in each IC is dominated by the information about the contralateral sound field (Jenkins and Masterton, 1982; Aitkin et al., 1984; Kelly and Kavanagh, 1994; Delgutte et al., 1999; Litovsky et al., 2002). An interaction between these two representations is mediated at the subcortical level by the commissure of the inferior colliculus (CoIC), a bundle of fibers that connects the two ICs and which constitutes one of the largest of the many afferent inputs the IC receives (Moore, 1988; Saldaña and Merchán, 1992, 2005).

The anatomical organization of the CoIC is well established, but we know relatively little about its functional properties (Adams, 1980; Coleman and Clerici, 1987; González-Hernández et al., 1991; Saldaña and Merchán, 1992; Malmierca et al., 1995, 2009). One of the problems in addressing this issue is the difficulty in breaking the connection the commissure makes between the ICs. Previous studies have attempted to do so by sectioning the CoIC, or by drug-induced blockade of glutamatergic transmission in one IC by pressure injection, while recording responses in the other (Moore et al., 1974; Malmierca et al., 2003, 2005). The first method is limited by the difficulty of the surgical procedure, uncertainty, prior to histological processing, that the commissurotomy is complete, and the impossibility of reversal. When applied *in vivo*, the second approach, while informative, yields a low number of tested units. This is primarily because of the difficulty in maintaining contact with a recorded neuron in the face of the mechanical instability caused by the pressure injection, and the time required for recovery from the drug effect (Malmierca et al., 2005). These limitations severely impede the testing of specific hypotheses about the function of the CoIC.

In an attempt to overcome these difficulties, we have tested the feasibility of using cooling-induced deactivation as a means of deactivating one IC in guinea pig. This means of neural deactivation, first developed at the beginning of the twentieth century [see Brooks (1983) for review], has been used extensively in both electrophysiological and behavioral studies, and has the important advantage of rapid onset and recovery (Schiller and Malpeli, 1977; Sherk, 1978; McClurkin and Marrocco, 1984; Girard and Bullier, 1989; Michalski et al., 1993; Payne et al., 1996; Lomber et al., 1999; Lomber and Malhotra, 2008; Girardin and Martin, 2009). It has been applied in several paradigms, including as a means of reversibly switching off the afferent input to a recorded brain region (e.g., Schiller and Malpeli, 1977; Sherk, 1978; McClurkin and Marrocco, 1984; Michalski et al., 1993; Nakamoto et al., 2008; Antunes and Malmierca, 2011).

An important question about the utility of the technique is the extent to which the cooling effect spreads through neural tissue, both from the perspective of ensuring that the tissue targeted for cooling has been fully deactivated, and that the recording site has not been functionally affected by spreading cold. These issues have been addressed in earlier investigations, but as cited above, mostly in the cortex, and mainly in cat and primate. Cooling has been applied to midbrain sites in only a few studies (e.g., Keating and Gooley, 1988; Lomber and Payne, 1996; Lomber et al., 2001, 2007b). In smaller animals such as guinea pig (Villa et al., 1999; Nakamoto et al., 2008; Coomber et al., 2011) and rat (Kayama et al., 1984; Yuan et al., 1986; Diamond et al., 1992; Villa et al., 1999; Antunes and Malmierca, 2011) cooling has only been used to deactivate cortex. Because several factors determine the efficacy of cooling (the geometry of the tissue, the surface area to which the cryoloop is applied, the blood flow in the tissue, and the distance between the sites of cooling and recording) it is not possible to extrapolate the effects of cooling from one brain structure to another.

Here we test the hypothesis that cooling-induced deactivation in guinea pig offers an effective means of deactivating one IC while recording sound-driven electrophysiological activity from the other IC. We discuss the extent to which the observed changes are mediated by commissural input rather than by descending pathways. We conclude that cooling can be used to abolish neuronal spiking in a well-defined part of one IC without deactivating the contralateral IC or neighboring nuclei, thus allowing us to observe the effects of substantially reducing commissural input.

## Material and methods

### Animals: maintenance and surgical preparation

All experiments were performed under the terms and conditions of a license issued by the UK Home Office under the Animals (Scientific Procedures) Act 1986 and with the approval of the Local Ethical Review Committee of Newcastle University.

Experiments were performed on 15 adult pigmented guinea pigs (*Cavia porcellus*) of either sex, weighing between 595 and 888 g. Additional data gathered in these experiments will be reported separately.

Anesthesia was induced with urethane (1 g/kg as 20% solution, i.p.) and supplemented by Hypnorm (1 ml/kg, i.m., VetaPharma, UK). Atropine (0.05 mg/kg, s.c.) was given following induction of anesthesia to suppress bronchial secretions. Anesthesia was maintained with further doses of Hypnorm (1/3 original dose) on indication of a pedal reflex elicited by a pinch to the hind paw. A tracheotomy was performed and an endotracheal tube inserted to maintain air flow.

Core temperature was monitored via a rectal thermometer and maintained at 38°C by a thermostatically controlled electric blanket (Harvard Apparatus). Animals were allowed to respire spontaneously, but if breathing became labored they were artificially respired with medical air via a modified small animal ventilator (Harvard Apparatus) to maintain the end-tidal CO_2_ at 5%.

Animals were placed in a sound attenuating room and the head secured in a stereotaxic frame in which the ear bars were replaced with hollow conical Perspex specula, the apices of which were placed in the auditory meatuses allowing visualization of the tympanic membranes.

A dorsal mid-sagittal incision was made along the length of the skull. The skin was reflected and the tissues overlying the skull were abraded. A small hole was trephined 10.5 mm caudal and 2.5 mm left of bregma. Rongeurs were used to extend the diameter of the craniotomy to 5 mm, centered on the pilot hole. To reveal the left IC, the dura was retracted and the overlying occipital cortex was aspirated with a glass Pasteur pipette attached to a vacuum pump (Matburn). The auditory thalamus and cortex were undamaged by this procedure.

### Electrophysiological recording

Electrical activity was recorded with glass-coated tungsten microelectrodes advanced into either the cooled or uncooled IC (Merrill and Ainsworth, 1972). Extracellular action potentials and local field potentials (LFPs) were amplified (x10,000) and filtered (0.1 Hz to 3 kHz) by a preamplifier (Dam-80; World Precision Instruments). The spike signal was further high-pass filtered (300 Hz) and amplified before being discriminated, converted to logic pulses, and time stamped to an accuracy of 10 μs by dedicated hardware (Tucker Davis Technologies, TDT System 2). As well as recording spike times, in some cases we also collected spike waveforms using a MacLab 4/e recorder (AD Instruments) running Scope software. This allowed us to monitor changes in the size and shape of spikes during the cooling cycles.

LFPs were recorded using the same electrode as the spike activity and extracted by low pass filtering the output of the preamplifier at 1 kHz, to remove spikes, before averaging the response using MacLab.

### Sound stimulation

Pure-tone, noise and click stimuli were generated by TDT System 2 hardware under computer control through custom-written software that allowed the frequency and level of stimuli to be varied in real time.

Stimuli were delivered by a calibrated closed acoustic system consisting of Sony MDR 464 earphones housed in an alloy enclosure and coupled to damped probe tubes (4 mm diameter) that fitted into the ear bars (Rees et al., 1997). The maximum output of the system was flat from 0.1 to 9 kHz (100 ± 5 dB SPL) and then fell with a slope of ~15 dB per octave. Second and third harmonic components in the signal were ≤60 dB of the fundamental at the highest output level.

On isolation of a single unit, the characteristic frequency (CF) and minimum threshold to contralaterally presented tones was determined audio visually to establish the settings for data collection.

Frequency response areas (FRAs) were derived as described previously to both diotic and contralaterally presented pure tones (Evans, 1979; LeBeau et al., 2001). The response area was constructed by counting the number of spikes elicited in response to 50-ms tone-bursts (5-ms rise/fall time, repetition rate 5/s), which varied in 51 logarithmically spaced frequency steps over an intensity range of 90 dB (in 5 dB steps). The number of spikes produced in response to each stimulus was counted and displayed color coded at the appropriate position in a plot of tone frequency versus attenuation level. Post stimulus time histograms (PSTHs) were constructed from the responses to 75-ms duration tones with a repetition rate of 4/s. LFP responses were recorded in response to transient 10-kHz tone pulses of 1-ms duration and 0.01 ms rise/fall time.

Recording location was determined by the presence of sound driven spiking and a tonotopic progression with electrode depth. All recordings were made within the IC from neurons with response properties that were similar to previous recordings made within the central nucleus (Rees et al., 1997; LeBeau et al., 2001), but we make no claims as to the exact location of individual recordings.

### IC cooling

**Figure 1:**
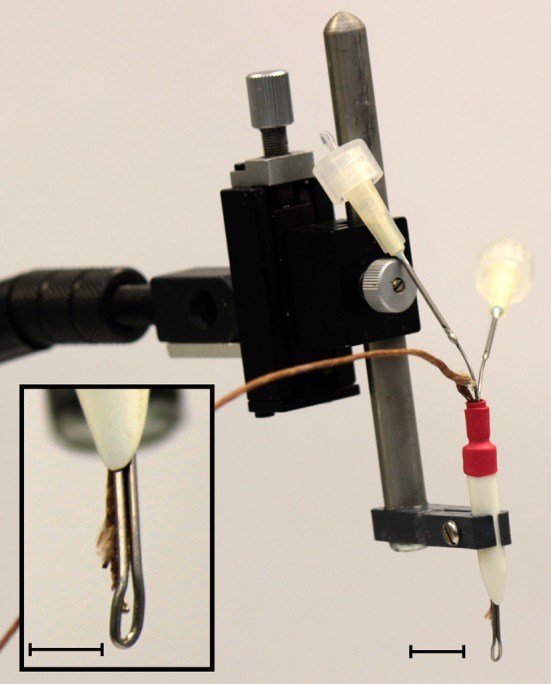
**Photograph of the cryoloop used to cool the IC held in a micromanipulator.** Two 19 gauge needles soldered to each end of the stainless steel cryoloop tubing acted as the inlet and outlet ports for the coolant. The manipulator allowed precise placement of the loop in contact with the exposed IC. Inset: detail of the cryoloop. The thermocouple tip allows precise monitoring of the cryoloop temperature. The surface of the cryoloop was placed in contact with the dorso-rostral part of the exposed IC. Scale bars = 5 mm.

Cooling of the IC was achieved using a variation of the method described by Lomber et al. (1999). A cryoloop was made by forming a loop from 23 gauge stainless steel tubing. Each end of the tubing was soldered to a 19 gauge needle (Figure 1). These needles served as the inlet and outlet for circulating coolant. A peristaltic pump (Gilson Minipuls 2) drew ethanol cooled in a deep freeze to −80°C from a reservoir. The ethanol was pumped through a coil of fluorinated ethylene propylene tubing (Cole-Parmer) inside a vacuum flask (Dilvac) containing ethanol cooled to −80°C before passing through the cryoloop and back into the reservoir. The tip of a type T (copper-constantan) thermocouple was secured to the cryoloop tip (Figure 1 inset) to allow monitoring of the cryoloop temperature with a digital thermometer (HH506RA, Omega). Regulating the pump speed allowed the flow rate through the system to be controlled thereby enabling the temperature at the cryoloop tip to be maintained at the desired temperature.

The cryoloop was curved to maximize contact with the dorso-lateral surface of the exposed IC. This placement was chosen to maximize the extent of cooling in areas in which the density of neurons projecting via the CoIC to the contralateral IC is greatest (Saldaña and Merchán, 1992; Malmierca et al., 1995, 2009). At no time was the temperature allowed to drop below 2°C to prevent cryo-trauma to the tissue. Temperature measurements within the IC were made using a needle thermocouple (HYP-0, Omega) that was advanced into the IC using a microdrive. In some cases the thermocouple was glued to a recording electrode so that temperature could be measured in the IC close to the site of neural recording.

Histological processing using standard methods followed by cresyl violet staining was performed in some experiments to reconstruct the penetrations of the thermocouple and in others to verify that cooling did not produce tissue damage.

## Results

The principal aim of this study was to establish whether a cryoloop can be used to cool one IC selectively and reversibly to deactivate spiking whilst electrophysiological recordings are made in the contralateral IC. To address this question we first measured the temperature in several sites in both ICs while cooling the left IC. Implicit in this aim is that there should be no functionally significant spread of cooling to the other IC or other brainstem auditory structures, and that cooling should not produce tissue damage.

### Temperature measurements in the cooled IC

**Figure 2:**
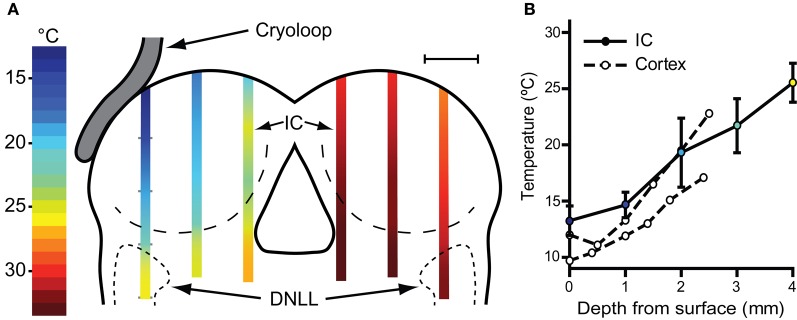
**(A)** Schematic coronal section through the IC showing placement of the cryoloop and temperatures measured in the IC with a needle thermocouple during cooling in the left (cooled) and right IC. These measurements were made after removal of the overlying cortex. For electrophysiological recording experiments the cortex over the right IC was left intact with the result that the temperature would be ~2°C warmer in the right IC (see text). **(B)** Mean ± SD of temperature measured in the lateral-most penetration of the left IC and three similar cases (filled circles, solid line). Open circles and dashed lines show temperature as a function of depth in guinea pig auditory cortex re-plotted from Coomber et al. (2011) for comparison.

With the cryoloop placed on the IC with no coolant flow, the *cryoloop* temperature was typically 32–35°C, a few degrees below the maintained core temperature of 38°C. To establish how effectively our cryoloop system cooled the IC we measured temperature along vertical penetrations aligned with the electrode tracks made to record neural activity. Cooling was begun and the cryoloop tip held at 5°C for approximately 10 min (Figure 2A) before the thermocouple was lowered from the dorsal surface of the exposed IC. Temperature measurements were taken along the dorso-ventral penetration at 1 mm steps measured from the surface. The most laterally placed penetration reached the dorsal nucleus of the lateral lemniscus (DNLL). The temperatures recorded are represented by the color gradients on the schematic coronal section in Figure 2A. The temperature as a function of depth from the dorsal surface of the IC for the most lateral penetration in the cooled IC is plotted in Figure 2B (filled circles and solid line). For depths less than 2–2.5 mm the temperature was <20°C. As well as a gradient in depth, there is also a gradient across the IC with temperatures in the most medial track being higher than those more laterally. For comparison, we have also re-plotted temperature measurements (open circles and dashed lines) from Coomber et al. (2011) taken at different depths in the guinea pig auditory cortex when the surface was cooled to 2°C. The temperature gradient with depth is qualitatively similar in the two models.

### Temperature measurements in the contralateral IC, cochlear nucleus and cochlea

The possible spread of cooling from the cooled IC to its contralateral counterpart was also assessed and the temperatures recorded in three penetrations in mirror image positions to those in the cooled side were measured (Figure 2A). At 1 mm below the surface the mean temperature recorded was 30.3 ± 0.9°C and increased with depth to 32.8 ± 0.60°C (*n* = 3) 4 mm from the surface. As in the left IC the temperature was lowest on the lateral side where 1 mm below the surface it was 28°C.

**Figure 3:**
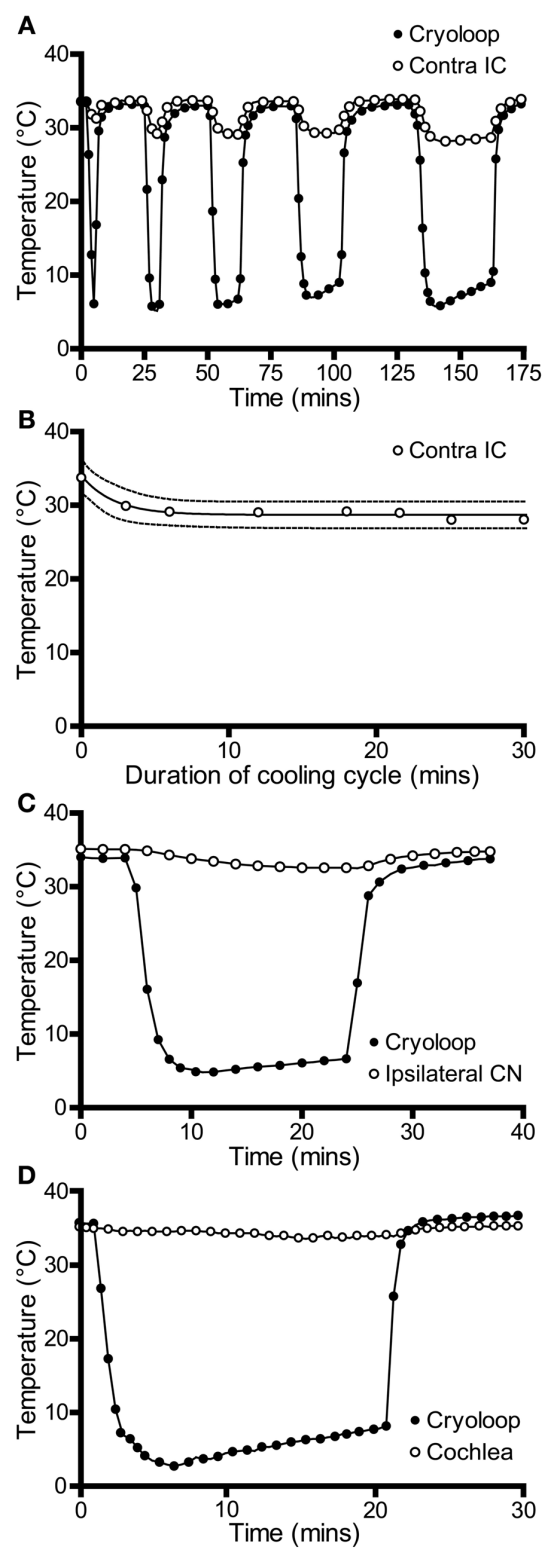
**(A)** Temperature of the cryoloop (filled circles) and at 1-mm depth in the contralateral IC (open circles) during repeated cycles of cooling of different duration. The temperature in the contralateral IC falls only a few degrees below control temperature. **(B)** Temperature in the contralateral IC (open circles) is relatively stable as the duration of the cooling cycle is increased progressively to 30 min. **(C)** Cooling the IC resulted in a less than 2°C reduction in temperature in the ipsilateral cochlear nucleus (CN, open circles) and **(D)** in the ipsilateral cochlear duct (open circles).

We also recorded temperatures in the right IC as a function of time over the course of the cooling cycle in three animals. The thermocouple tip was placed 1 mm below the surface of the contralateral IC in the mirror image position to that used to measure the IC cooled by the cryoloop. In each case the cryoloop temperature was reduced to ~5°C and the temperature was measured in the contralateral IC after the temperature had stabilized. The lowest absolute temperatures measured in the contralateral IC ranged from 30.7 to 28.1°C, with a mean reduction of 4.3°C from control. The effect of varying the duration of cooling on the temperature in the contralateral IC was assessed by applying five cooling cycles which varied in duration between 0.5 and 30 min with the cryoloop temperature held at ~5°C in each case (Figure 3A). The reduction in temperature in the contralateral IC ranged between 3.7°C after 3 min of cooling and 5.4°C after 30 min of cooling. The minimum temperature in each cooling cycle plotted as a function of cycle duration was fitted with a single phase exponential decay function (*r*^2^ = 0.96; Figure 3B). These values demonstrate that the temperature of the IC remained stable over periods up to 30 min of cooling the contralateral IC.

The measurements described above were made with the cortex overlying the IC removed to facilitate placement of the thermocouple. In some cases we also compared temperature in the right IC contralateral to the cooled side before and after aspiration of the cortex. These measurements were made by introducing the thermocouple into the IC through the cortex using mirror image coordinates to those used for placement in the cooled IC. After making measurements the cortex was aspirated with the thermocouple in place and the measurements repeated and the recording positions relative to the right IC confirmed. The temperature measured 1 mm from the surface of the IC was ~2°C warmer with the cortex intact indicating that the cortex served to insulate and warm the IC. At larger depths the difference in temperature was reduced to ~0.5°C.

We also measured the effect of cooling the IC on the temperature of two other structures in the auditory pathway: the cochlear nucleus and the cochlea. In both cases measurements were made from the structures ipsilateral to the cooled IC. This was done because we predicted that these structures were most likely to be affected by cooling and they provide the predominant input to the contralateral IC—the ultimate target of our electrophysiological recordings. The temperature of the cochlear nucleus never fell more than 2°C below control throughout 20 min of cooling (Figure 3C).

The temperature of the cochlea was measured in three ways. When the thermocouple tip was placed in contact with the round window, the temperature fell by 1.8°C during cooling (Figure 3D). A second set of measurements were made with the thermocouple placed on the first turn of the cochlea, and finally a small hole was made in the bony wall and the thermocouple tip placed inside the cochlea. In each case the temperature drop measured was <2°C from the control temperature (33.7°C) after the loop had been cooled to ~5°C for 20 min.

### Histological analysis of cooled tissue

**Figure 4:**
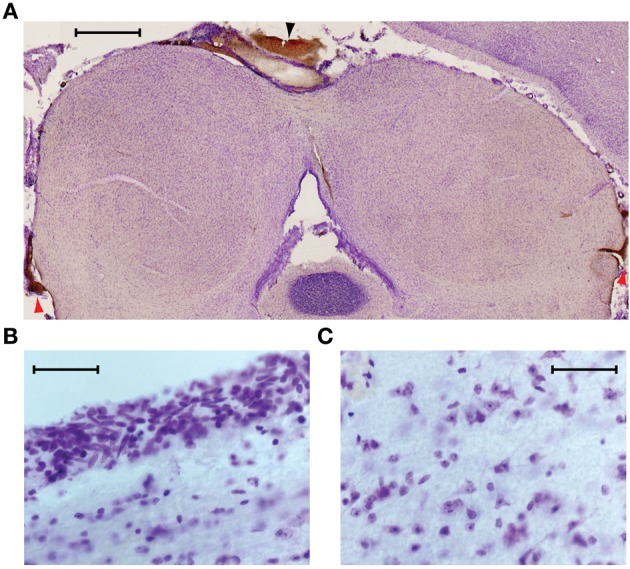
**(A)** Near coronal section through the IC following an experiment. Aspiration of the cortex overlying the left IC and placement of the cryoloop did not produce any noticeable trauma to the tissue. Blood at the midline (black arrowhead) and bilaterally at the ventro-lateral edges of the tectum (red arrowheads) resulted from aspiration of the cortex. Scale bar = 1 mm. **(B)** Neurons near the dorsal surface of the IC contacted by the cryoloop show no sign of damage. **(C)** Neurons within the cooled IC have normal morphology and no signs of ischemia are present. Scale bar in **(B)** and **(C)** = 50 μm.

We used histology to assess the integrity of the neural tissue after its exposure to temperatures of ~5°C. In six experiments in which the IC underwent multiple cooling cycles over the course of several hours, the animal was perfused and sections were cut through the IC at 50 μm thickness to check for signs of cryogenic tissue damage or ischemia. Figure 4 shows a transverse section through the IC, cut approximately midway through the rostro-caudal axis, and stained with cresyl violet. The cortex that normally overlies the left IC was aspirated to allow placement of the cryoloop. In this, and the other cases, the shape and structure of the cooled IC appeared normal. There was some bleeding from the edges of the damaged cortex that formed small clots along the midline (black arrowhead) and at the ventro-lateral edges of the tectum, lateral to each sagulum (red arrowheads). There were no clots on the surface between the cryoloop tip and the IC, and no sign of vascular damage within the tissue.

Higher magnification images taken from the dorsal surface (Figure 4B) and the center of the cooled IC (Figure 4C) show cells with normal morphology and intact nucleoli. Comparison with tissue in the uncooled IC shows no discernible differences.

### Neural activity in the cooled IC

The effect of cooling on sound-driven neural activity was assessed by recording single units and multi-unit clusters at different depths in the cooled IC. Recordings were completed for control, cool, and recovery phases from 33 single units and 9 multi-unit clusters, in 11 experiments. The responses to multiple repetitions of a pure tone 20 dB above threshold at CF were collected in each condition. To test for any change in firing rate, the number of spikes evoked per stimulus in each condition was assessed with a Friedman omnibus test. Multiple Wilcoxon signed ranks tests were used to determine differences between pairs of values between the three conditions. In order to be included in these analyses, the control and recovery data had to show no significant difference in the number of spikes per stimulus (*p* > 0.017, following Bonferroni correction) and the PSTH had to recover the same pattern as seen in the control condition. Of the 33 single units, 21 passed this criterion and are included here. The firing rate in the recovery condition of single units that reached criterion ranged from 72.0 to 127.6% of control (median = 95.2%; interquartile range (IQR) = 88.8–100.6%). Of the 9 multi-unit clusters, 7 passed the criterion for inclusion (median recovery firing rate = 98.7%; IQR = 85.0–106.6%; range = 71.5–121.3%).

The minimum cryoloop tip temperature differed for each single neuron to maximize the chances of holding the unit to obtain recovery data. If a neuron showed a marked reduction in firing rate, cooling was discontinued. The lowest cryoloop tip temperature was 2°C while the highest maximal cooling temperature applied was 20.3°C (median = 8.1°C). The firing rates of all included units were not normally distributed (D'Agostino-Pearson omnibus K2 test). A Friedman test showed a statistically significant difference in firing rate between the control, cool and recovery groups of all units (χ^2^(2) = 53.5, *p* < 0.001). Wilcoxon Signed Ranks Tests found significant differences in the control versus cool (*Z* = −5.6, *p* < 0.001) and cool versus recovery conditions (*Z* = −5.4, *p* < 0.001) but no significant difference in the control versus recovery conditions (*Z* = −1.6, *p* = 0.11).

### Changes in neural activity as a function of temperature

**Figure 5:**
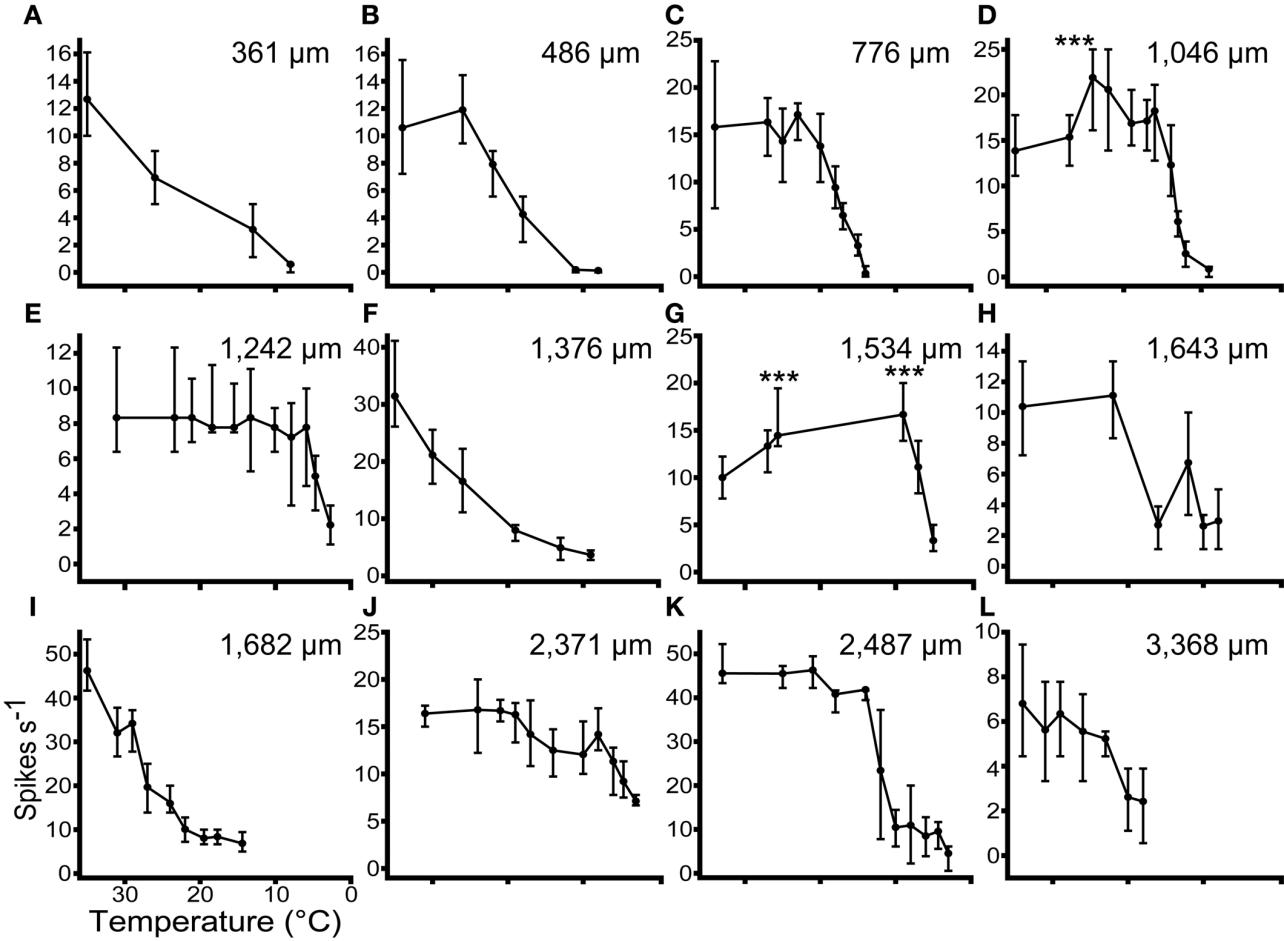
**The responses (Median ± IQR) of 12 single units to multiple presentations of a pure tone at CF, 20 dB re threshold, during stepwise cooling of the IC.** Units were recorded along the dorso-ventral axis of the IC from 361 μm **(A)** to 3368 μm **(L)** from the dorsal surface. Cooling into the range 10–20°C induced a significant (*p* < 0.001) reduction in firing rate in all 12 units. In 2 units **(D** and **G)** firing rate increased significantly re control (*p* < 0.001) prior to decreasing at lower temperatures.

In 17 units we recorded tone driven responses while progressively cooling the IC. Figure 5 shows the effect of cooling on the firing rate of 12 single neurons ordered by their depth in the IC. The effect of temperature on firing rate follows one of three patterns. In the majority of cases firing rate remained relatively constant and then decreased as the temperature was reduced further (Figures 5B,C,E,H,J,K,L). A second group showed a similar decline in firing rate when the temperature fell below 20°C, but this was preceded by an elevation in firing rate when the temperature was in the range 20–26°C (e.g., Figures 5D,G). In a few units the firing rate began to decline immediately after the temperature was reduced (e.g., Figures 5A,F,I).

The occurrence of these types does not follow any specific pattern with recording depth. Across all the units tested with stepwise cooling, 4/17 (24%) showed a statistically significant increase in firing and 3/17 (18%) showed a reduction in firing at temperatures in the range 20–30°C. The remainder showed no statistically significant change over this temperature range. As the temperature was further reduced all units showed a statistically significant reduction in firing rate.

**Figure 6:**
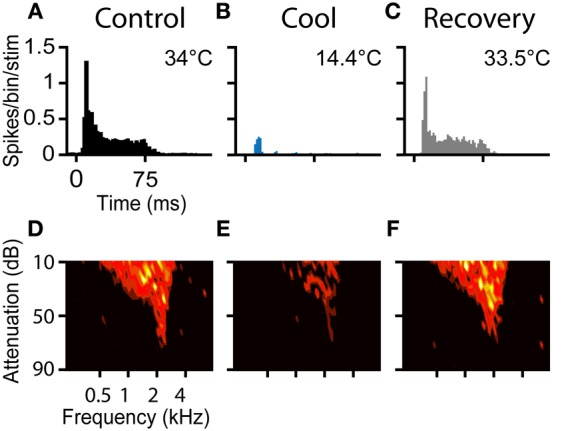
**(A–C)** PSTHs and **(D–F)** FRAs during control, cool to 14.4°C, and recovery conditions, respectively for a unit with CF = 2.1 kHz. The unit had an on-sustained PSTH and a V-type FRA under control conditions. During cooling only an occasional spike near stimulus onset remained, but the frequency tuning was retained. On recovery the unit regained its original PSTH shape and firing as a function of frequency returned to control levels.

The PSTH and FRA of a neuron in response to tones before during and after cooling are shown in Figure 6. The neuron was characterized in the control condition as an “on-sustained” type (Figure 6A) with a V-shaped response area (Figure 6D) according to criteria defined previously (Rees et al., 1997; LeBeau et al., 2001).

Prior to cooling, the neuron fired 753 spikes in response to the 100 presentations of the PSTH stimulus (median = 8, IQR = 6–8). Maximal cooling resulted in the number of recorded spikes falling to 103 (median = 1, IQR = 0–2) with onset spikes accounting for the majority. Cessation of cooling produced an immediate recovery of spiking activity. When the temperature measured at the cryoloop tip returned to near the control value (33.5°C), the neuron fired 757 spikes in the PSTH paradigm (Figure 6C). In addition to the recovery in spike count, the shape of the PSTH returned to its original on-sustained form. Firing rate was modulated by cooling in a similar manner in response to monaural or binaural stimulation (not shown).

A Friedman test showed there was a statistically significant difference between the firing rates across the control, cool and recovery conditions (χ^2^(2) = 150.4, *p* < 0.001). *Post-hoc* analysis with Wilcoxon Signed Ranks Tests found a statistically significant difference between the control and cool (*Z* = −8.6, *p* < 0.001), and cool and recovery (*Z* = −8.6, *p* < 0.001) conditions, but no significant difference between the control and recovery conditions (*Z* = −0.38, *p* = 0.71).

The FRA of the unit had a threshold at 75 dB attenuation and a CF of 2.1 kHz, with a Q_10_ of 3.40 and a Q_40_ of 1.00 (Figure 6D). The unit fired to 173 of the 936 frequency-level combinations presented. When cooled the number of frequency-level combinations that elicited spikes fell to 87 (Figure 6E). Cooling increased the Q_10_ to 4.38 and the Q_40_ to 1.44, although throughout all stages of cooling, threshold and CF were unchanged. On recovery, the neuron regained tuning and firing rate properties similar to those in the pre-cooled condition (Figure 6F). The area of the FRA driven by sound increased to 182 bins with a concomitant reduction in Q_10_ to 3.40 and Q_40_ to 1.19.

### Neural deactivation by cooling as a function of depth in IC

**Figure 7:**
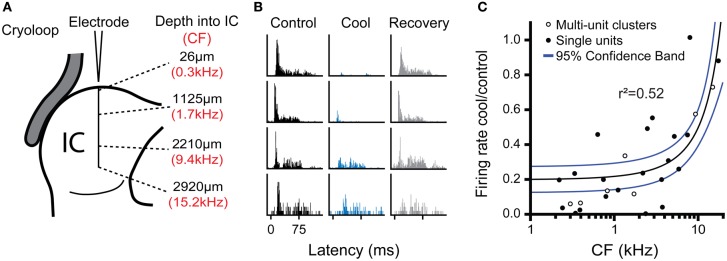
**(A)** Schematic representation of multi-unit recordings made at approximately 1 mm steps in an electrode penetration along the dorso-ventral axis of the IC showing depth from the dorsal surface (black text) and CF of the recorded cluster (red text). **(B)** PSTHs for activity recorded at the four locations shown in **(A)** during control, cool, and recovery. Cooling resulted in a notable reduction of spiking in the three most dorsal positions, but had less effect in the deepest position. **(C)** Ratio of firing rate in the cool and control conditions as a function of unit CF for single and multi-unit cluster recordings pooled across experiments. The reduction in firing with cooling was most evident for low CFs (dorsal locations), while neurons with higher CFs in more ventral locations were less affected. Regression line (black) and 95% confidence limits (blue).

To estimate the neural deactivation of the IC as a function of depth, we measured the responses of neurons at different frequencies and depths in the IC during cooling. To increase the sample size in this analysis we also sampled multi-unit clusters with the same procedure as for single unit recordings, except two or three neurons were recorded simultaneously. The responses of multi-unit clusters were modulated by cooling in similar manner to well-discriminated single units. Figure 7 shows examples of data taken from one experiment where neural activity was recorded at approximately 1 mm intervals along a single electrode penetration following the dorso-ventral axis of the IC (Figure 7A). The deviations from exact 1 mm steps along the track were to maximize the amplitude of the spike response in each position. The CF of the activity along the track followed the established tonotopic sequence with neurons tuned to low frequency dorsally and CF increased with depth.

All four locations were recorded with the cryoloop in the same position during cooling cycles of approximately 10-min duration with the cryoloop temperature held at 5 ± 3°C throughout. Cooling induced a gradient of neural deactivation along the track as revealed by the responses to monaural stimulation of the contralateral ear (Figure 7B) and similar responses to diotic stimulation (not shown). For the two most superficial positions firing rate was almost totally abolished, while for the two deeper positions there was a substantial reduction in firing in both cases.

Freidman's tests showed there was a statistically significant difference in firing rate between the control, cool, and recovery conditions in the three dorsal-most positions (26 μm: χ^2^(2) = 73.2; 1125 μm: χ^2^(2) = 93.5; 2210 μm: χ^2^(2) = 26.0; all *p* < 0.001). In the ventral-most position there was no significant difference in firing rate across groups (χ^2^(2) = 1.10, *p* = 0.58) despite a change in the PSTH morphology. *Post-hoc* analysis with the Wilcoxon Signed Ranks test for the dorsal-most position showed a statistically significant difference between the control and cool (*Z* = −8.0, *p* < 0.001) and the cool and recovery conditions (*Z* = −6.0, *p* < 0.001), but no significant difference between the control and recovery conditions (*Z* = −1.1, *p* = 0.27). The same pattern was found in the responses at 1125 μm from the dorsal surface. There was a significant difference between the control and cool (*Z* = −8.4, *p* < 0.001) and cool and recovery (*Z* = −7.5, *p* < 0.001) conditions, but no significant difference between the control and recovery conditions (*Z* = −1.7, *p* = 0.082). In the third position, the difference between the control and cool values had a lower Z statistic, but was still significant (*Z* = −5.0, *p* < 0.001). The same was true of the cool and recovery conditions (*Z* = −5.1, *p* < 0.001), with no significant difference between the control and recovery conditions (*Z* = −1.9, *p* = 0.06). In the ventral-most location there were no significant differences between any of the conditions.

Figure 7C shows the ratio of firing in the cool and control conditions for all the data collected in this analysis plotted as a function of the CF of the recording position. The maximal reduction in firing rate during cooling was fitted with a linear regression against CF (*r*^2^ = 0.52, *p* < 0.001). The fit indicates that the cooling produced by the cryoloop in our configuration produced a 50% or greater reduction in firing rate for locations representing CFs up to 6–8 kHz.

**Figure 8:**
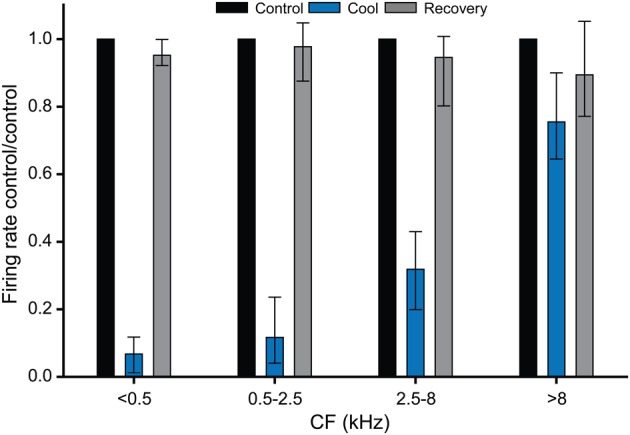
**Histograms showing the effect of cooling induced deactivation for single units grouped by CF.** All units with a CF ≤ 8 kHz showed a statistically significant reduction in firing on cooling re their control (pre-cool) values, whereas units with CF >8 kHz did not show a statistically significant reduction in firing during cooling. In all cases firing rates recovered close to control values on rewarming.

Figure 8 summarizes the data collected for the effects of cooling on firing rate in the cooled IC, plotted in four frequency ranges. This plot confirms that cooling was effective in deactivating neural activity at CFs up to ~8 kHz. For units with CFs greater than 8 kHz there was no significant difference between the firing rates in the control and cooled conditions. Temperatures measured at the depths in the IC at which the units were recorded indicate that cooling to below 25°C was necessary to produce a significant reduction in firing rate. This graph also demonstrates the recovery of firing rate after cooling was terminated and the tissue was allowed to rewarm.

### CF was unchanged by direct cooling

FRAs were recorded under control, cool and recovery conditions from 14 single units and 2 multi-unit clusters in this sample. CFs ranged from 0.18 to 6.36 kHz in the control condition. Except for one unit in the cool and another unit in the recovery condition, the unit CFs were not substantially changed by the paradigm. During cooling, the median change was 1, with an IQR of 0.97–1.05, and a range of 0.86–1.24. Following recovery, the median change was again 1, the IQR was 0.93–1.08, and the range was 0.86–1.22. The largest change observed was an increase in CF from 1.7 to 2.1 kHz during cooling. This unit had a closed FRA and a small change in firing rate produced the 24% change in CF. For the population, a Friedman test found no significant difference between CF in the control, cool, or recovery conditions (χ^2^(2) = 0.84, *p* = 0.66).

### Effects of cooling the IC on neural responses in the contralateral IC

After establishing the effects of cooling on neural activity in the IC, the second aim of this paper is to demonstrate its viability as a means of studying the influence of the IC on neural processing by its contralateral counterpart. Here we present examples of data in the form of single unit activity and LFPs showing the effects of cooling in the contralateral IC.

**Figure 9:**
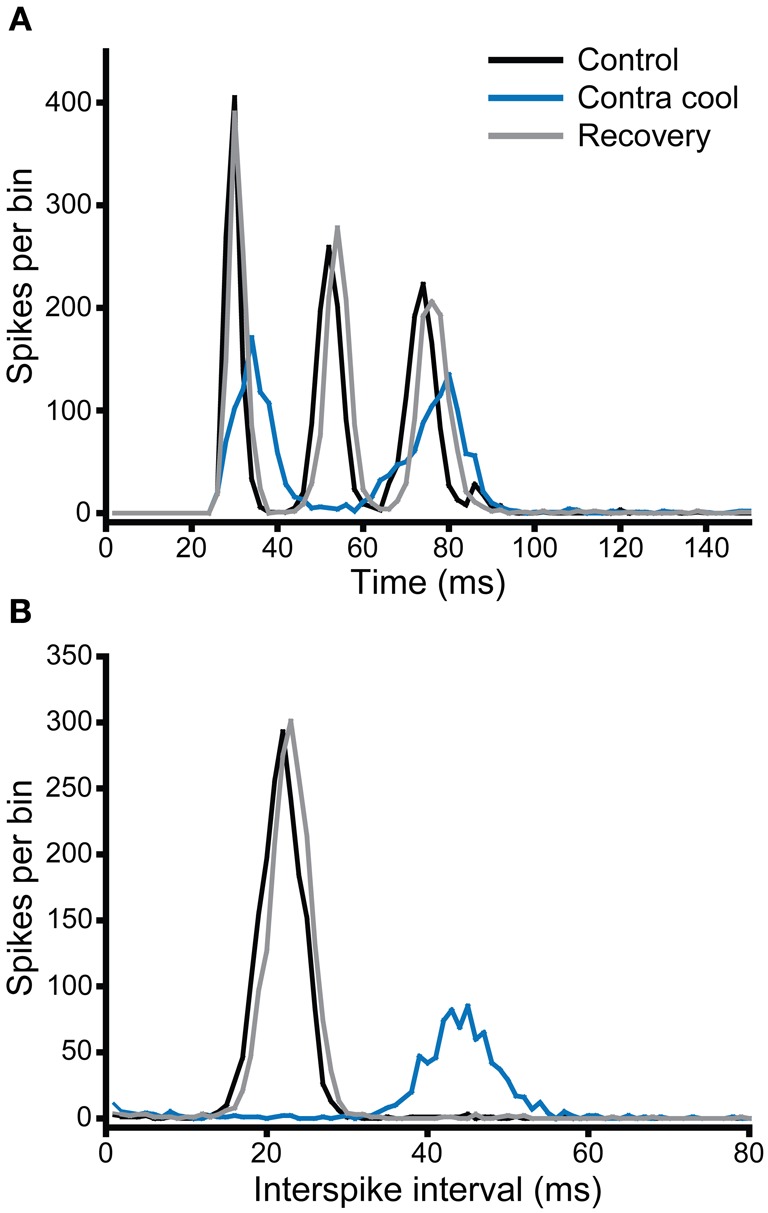
**(A)** PSTH and **(B)** ISIH for a unit with a chopper response (CF = 1.1 kHz) that showed a change in firing rate and temporal pattern (mediated by an increase in ISI) during cooling of the contralateral IC. Both rate and ISI values recovered on re-warming to control temperature.

Figure 9A shows the PSTH of a single unit, which under control conditions (black) had a chopper response with three clearly identifiable peaks indicating a regular firing interval of ~22 ms. This is confirmed by the single narrow peak centered on 22 ms in the interspike interval histogram (ISIH, Figure 9B). When the contralateral IC was cooled, the PSTH (blue) showed an overall reduction in the number of spikes per stimulus, as well as a notable increase in the latency to the first peak, a reduction in the number of peaks from three to two, and less regular firing exemplified by the lower and broader peaks in the PSTH. This is confirmed by the shift in the peak of the ISIH to a longer interval (~44 ms) and the reduction in regularity is indicated by the lower height and broader width of the peak. When cooling was stopped, and the IC returned to near control temperature, the changes seen with cooling were reversed (gray).

**Figure 10:**
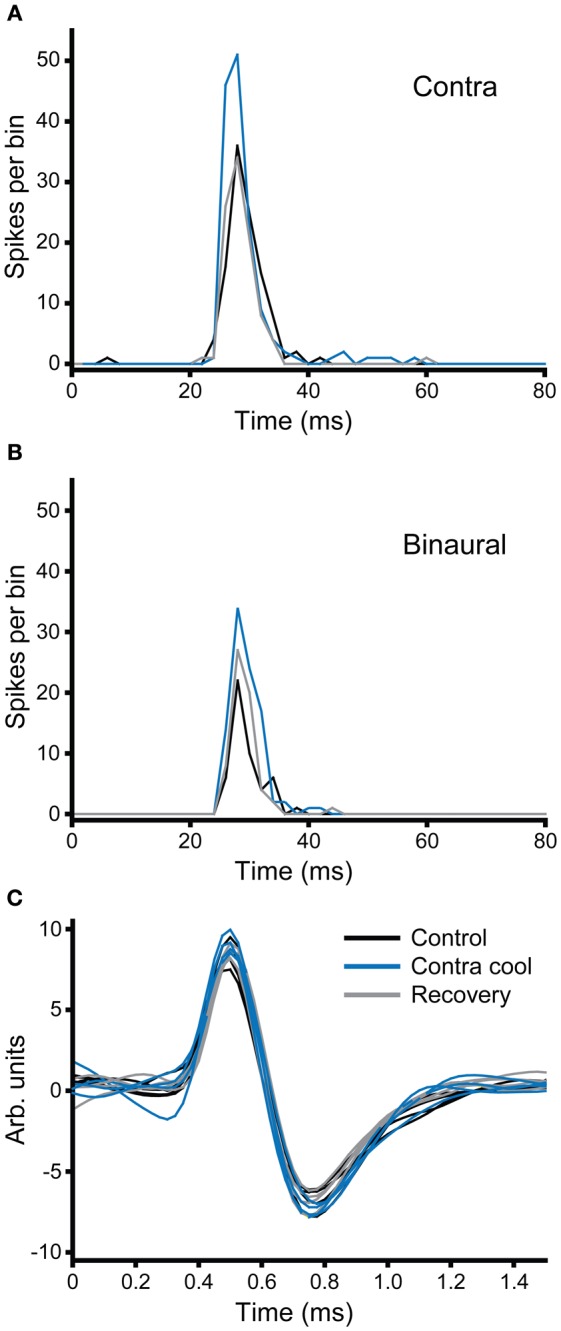
**(A,B)** PSTHs of a broad-onset unit (CF = 8.1 kHz) recorded in the IC contralateral to the cryoloop before, during and after cooling. The number of spikes elicited to stimulation of the contralateral ear **(A)** increased during cooling and returned to the control value on recovery. In response to binaural stimulation **(B)** the unit showed an EI characteristic, but firing similarly increased with cooling. **(C)** Five examples of action potentials recorded during each of the three stages of the paradigm; the morphology of the action potential did not change during cooling of the contralateral IC.

In the second example illustrated in Figure 10, the effect of cooling is demonstrated under two different stimulus conditions: monaural contralateral stimulation (Figure 10A) and binaural diotic stimulation (Figure 10B). Under control conditions the unit fired fewer spikes with binaural stimulation indicating that it had an excitatory-inhibitory (EI) type response (black). In both conditions cooling the IC increased the firing rate (blue). The increase in firing rate was greater for contralateral stimulation, but the relative increase under both conditions was about the same. When the IC rewarmed, the changes observed with cooling were reversed (gray), but this was more complete for the case where stimuli were presented to the contralateral ear. Figure 10C shows examples of the action potential waveforms for this unit, color coded as for the PSTHs. The shape, size, and time course of the waveform remained the same in control, cool, and recovery conditions. The absence of any change in the action potentials makes it unlikely that the changes observed in the firing rate of the unit are attributable to temperature change in the recorded IC (Volgushev et al., 2000b; Cao and Oertel, 2005).

**Figure 11:**
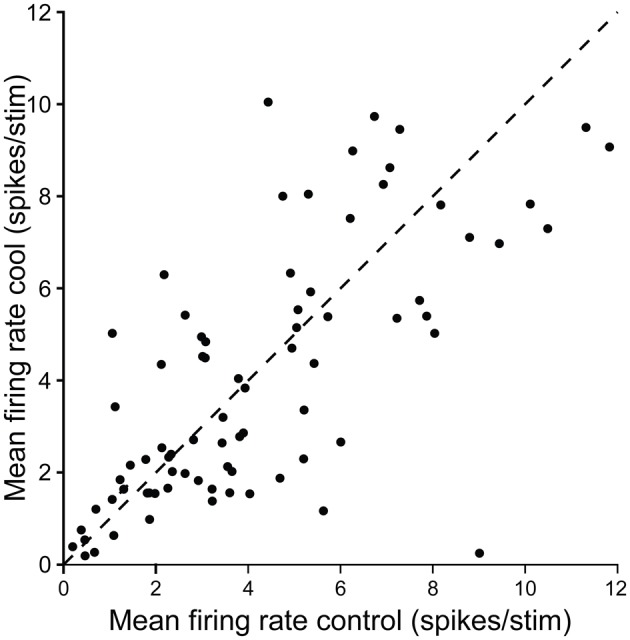
**Scatter plot showing control firing rate versus firing rate during cooling for single neurons recorded in the IC while cooling the contralateral IC.** Dashed line shows the line of identity.

Figure 11 summarizes the changes in firing rate for a population of single units in the IC contralateral to cooling in response to repeated stimulation with a tone at their CF. Cooling led to an increase or decrease in firing rate in different neurons, and although the degree of change varied considerably between units in some cases it was substantial.

**Figure 12:**
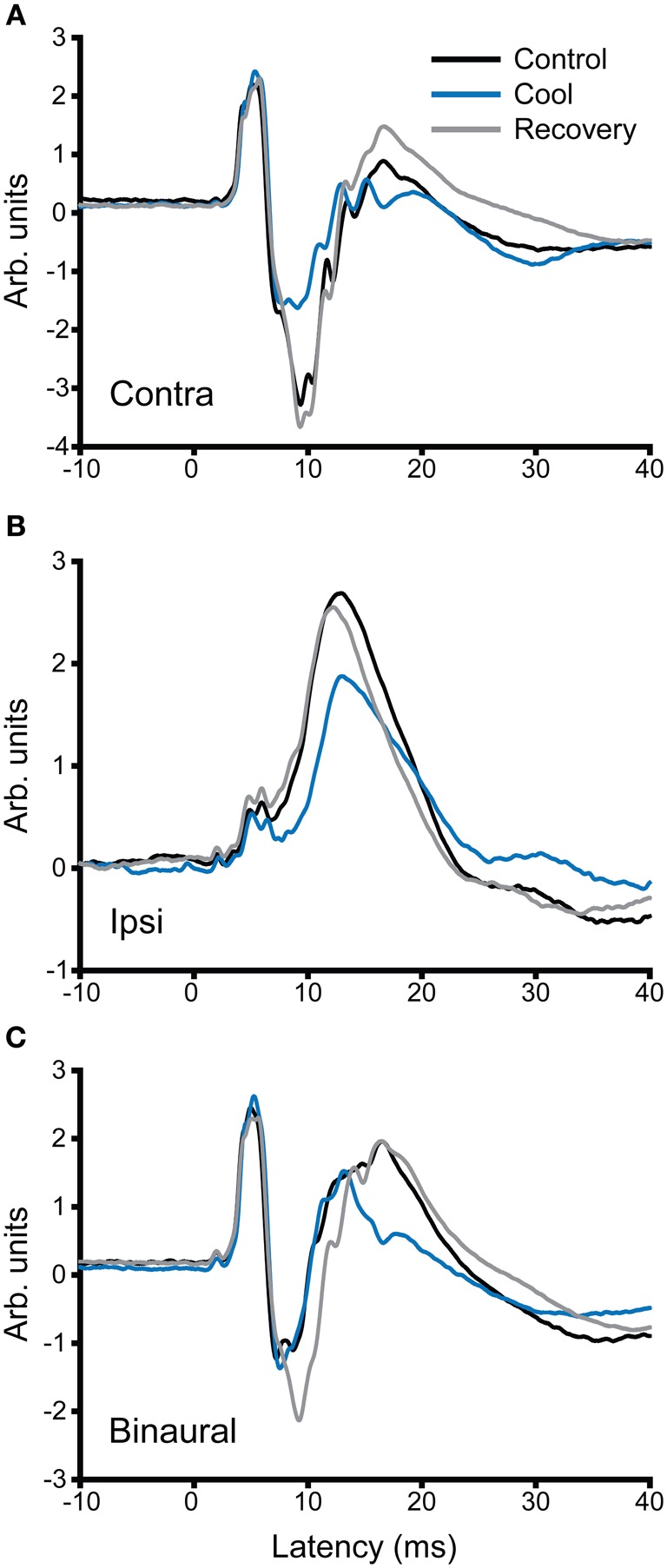
**Averaged local field potentials (100 sweeps) recorded in the IC in response to stimuli (1-ms duration tone pulses) presented at time zero to (A) the ear contralateral to the recorded IC, (B) the ipsilateral ear, and (C) binaural stimulation.** Responses are shown for control conditions (black), during cooling (blue) and rewarming (gray) of the contralateral IC. Cooling has little effect on the first peak in the waveform, but changes the amplitude and time course of components occurring at and after 10 ms post stimulus onset.

In addition to recording single unit activity, we assessed the impact of cooling the contralateral IC on the LFP. Figure 12 shows examples of LFPs recorded from the IC before, during and after cooling of the contralateral IC under three stimulus conditions, contralateral, and ipsilateral monaural stimulation (Figures 12A,B, respectively) and diotic stimulation (Figure 12C). In the case of contralateral and binaural stimulation there was a pronounced upward-going component with a post-stimulus latency of ~6 ms that corresponds to the afferent volley of activity to the IC followed by a biphasic waveform that returned to baseline at ~30 ms. The afferent volley was less pronounced in the case of the response to ipsilateral stimulation. When the contralateral IC was cooled the magnitude and latency of the afferent volley was unchanged, but there were clear changes in the later potentials. The form and extent of the changes varied between the different stimulus conditions. In the case of contralateral stimulation the largest change occurred in the first negative-going potential whereas with diotic stimulation the largest difference between cool and control was in the positive-going potential at ~17 ms. With ipsilateral stimulation the single broad positive waveform was delayed, diminished in height and became broader.

## Discussion

Cooling has been demonstrated to be an effective means of deactivating brain tissue in many reports where it has been adopted for behavioral or electrophysiological studies (Schiller and Malpeli, 1977; Sherk, 1978; Girard and Bullier, 1989; Michalski et al., 1993; Payne et al., 1996; Lomber et al., 1999; Lomber and Malhotra, 2008; Girardin and Martin, 2009). The nature of the technique, however, requires that it be validated in each situation to which it is applied. The most important considerations are first that it is effective in deactivating the targeted center in a reversible manner, and second that the cooling effect is sufficiently restricted that it does not impact on other centers involved in the process under investigation. Our aim here was to establish the utility of cooling as a method for discovering how one IC is influenced by the other. If the technique can be demonstrated to be viable it would confer considerable advantages over the drug injection techniques used previously for this purpose (Malmierca et al., 2003, 2005). Our findings show that cooling the IC can fulfill both the required criteria.

Cooling has been applied to the auditory cortex in behavioral studies and to study its interactions with the medial geniculate body and the IC (Villa et al., 1991; Lomber and Malhotra, 2008; Nakamoto et al., 2008; Coomber et al., 2011), but it has not previously been applied directly to the IC itself. Indeed, the only previous reports of the application of cooling in the midbrain investigated superior colliculus function in cat and macaque both of which have a larger brain than guinea pig (Keating and Gooley, 1988; Lomber and Payne, 1996; Lomber et al., 2007b). Furthermore, in these studies the effect of deactivation was tested on behavior rather than on direct measures of neuronal function.

Our results demonstrate that spiking activity in the IC can be almost abolished only a few minutes after cooling begins and activity returns to control values within a few minutes of its termination, with in some cases an overshoot in activity following rewarming. Cooling was not homogenous throughout the depth of the IC. A temperature gradient extended along the dorsal to ventral axis of the IC corresponding to its well-established tonotopic gradient (Rose et al., 1963; Merzenich and Reid, 1974; Semple and Aitkin, 1979; Malmierca et al., 1995). Cooling appears to be effective to a depth of about 2 mm into the IC (Figure 7) when the cryoloop is cooled to 2–5°C. Cooling to this depth was sufficient to deactivate neurons representing frequencies up to 6–8 kHz. Although this covers only part of the auditory range, which extends to ~50 kHz in the guinea pig (Heffner et al., 1971; Prosen et al., 1978), it encompasses key frequency ranges such as those over which interaural time and interaural level difference mechanisms dominate for sound localization.

The efficacy of cooling in the IC is similar to the ~2 mm depth of deactivation reported when cryoloops were placed on the surface of the cerebral cortex in cat and guinea pig (Lomber and Payne, 2000; Lomber et al., 2007a; Girardin and Martin, 2009; Coomber et al., 2011). Intuitively, one might expect cooling to be more efficient in the IC since, in contrast to the relatively flat surface of the cortex in guinea pig, the exposed colliculus protrudes from the surrounding brainstem tissue, making a relatively larger part of its surface area accessible to contact with the cryoloop. On the other hand, the high blood flow in the IC might mitigate the cooling effect. The IC is highly vascular and demonstrates one of the highest blood flows of all brain regions and the vascularization is non-isotropic (Reivich et al., 1969; Gross et al., 1987; Dorr et al., 2007; Song et al., 2011). This high blood flow will warm the IC by convection and so counteract the cooling effect, and could also increase the rate of rewarming when the flow of coolant in the cryoloop circuit is stopped.

As reported by others, we found no anatomical evidence of tissue damage after cooling, even when the IC had been exposed to multiple cooling cycles to ~5°C (Keating and Gooley, 1988; Lomber et al., 1999; Yang et al., 2006; Girardin and Martin, 2009). Following histological processing, cells were intact and there was no obvious difference between stained tissue from cooled and uncooled ICs. We were careful never to allow the cryoloop temperature to fall below 2°C and it is likely that even the tissue in immediate contact with the cryoloop would be higher than the cryoloop temperature. Consistent with the histological evidence, we found that in almost all cases the functional properties of the neurons in the cooled IC recovered to control values when the tissue was allowed to rewarm. In some cases there was rebound of neuronal firing, such that immediately after cooling was stopped firing rates temporarily exceeded control values.

Cooling had a profound effect on neuronal firing rate in the cooled IC. This is evident from several measures of neuronal function, including average firing rate and temporal firing patterns as represented by the PSTHs and FRAs. Although neuronal firing rates were significantly reduced by cooling, it was typical that neurons retained CFs similar to control (e.g., Figures 6D–F). This is consistent with the notion that cooling affects the synaptic efficacy of the afferent input to neurons, but stimulus selectivity is retained. A similar reduction in firing or synaptic potential, but with retention of stimulus selectivity has been observed for orientation tuning of neurons in visual cortex in cat (Michalski et al., 1993; Ferster et al., 1996; Girardin and Martin, 2009).

With a few notable exceptions (e.g., Michalski et al., 1993), most previous studies that employed cooling as a deactivation technique did not quantify the changes in response during the initial stages of cooling. Our results show that when the IC was progressively cooled all neurons showed a reduction in firing rate when the cryoloop temperature fell below about 20–10°C, consistent with previous reports (Michalski et al., 1993; Lomber et al., 1994, 1999), but three different patterns are apparent in the response over the first 10–15° of cooling. Most neurons show little change in firing rate over this range, but 24% show an elevation in firing rate and 18% show a notable decline in firing rate for temperatures below 30°C (Figure 5). These patterns did not correlate with recording depth in the IC so it is unlikely the effects are a consequence of the location of the neuron in the IC, rather it suggests that some neuronal types may be more sensitive to cooling than others. As well as revealing the direct influence of cooling on individual neurons, such effects may also demonstrate the consequences of cooling in local networks. For example, a possible explanation for the severe reduction in firing with moderate cooling exhibited by some neurons is that by increasing the activation of inhibitory interneurons, moderate cooling leads to the more pronounced inhibition of neurons that receive this inhibitory input.

Biophysical studies, mainly *in vitro*, which have investigated how cooling influences neuronal responses show that it decreases synaptic efficacy by reducing the probability of transmitter release, and it has a differential effect on the operation of ion channels (Benita and Conde, 1972; Hardingham and Larkman, 1998; Volgushev et al., 2000a,b; Trevelyan and Jack, 2002; Cao and Oertel, 2005). At temperatures where neurons become virtually deactivated (~10°C), firing fails as the biophysical changes induced by cold induce depolarization block although firing may still occur if the stimulus is sufficiently strong (Volgushev et al., 2000b). The progressive increase in membrane potential occurs as the conductances of passive and voltage-gated potassium channels are decreased while sodium conductances remain relatively unaffected. Such changes are consistent with our observations that some spikes occur even at very low temperature. At temperatures above the level of deactivation, in the range of 18–24°C, cooling may lead to an increase in excitability (e.g., Volgushev et al., 2000b). As a consequence, these authors pointed out that during cooling there may be a penumbra of tissue around the deactivated region in which neurons show elevated firing. Such effects are consistent with the elevation in firing rate above the uncooled value we observed in some neurons when the temperature was between 20 and 26°C. The consequences of this effect need to be borne in mind when interpreting cooling experiments. A possible advantage of the IC as a target for the technique is that it is organized as a stack of frequency band laminae in which interconnections along the dorso-ventral axis are less evident (Malmierca et al., 1995). This is in contrast to the cortex where processing depends on micro circuits involving neurons and connections distributed between the layers.

A key factor in determining the utility of cooling for deactivating the IC is the extent to which deactivation is limited to the cooled IC. Our measurements suggest that the temperature of structures neighboring the cooled IC are not significantly reduced by cooling the IC. Our measurements show that the ventral part of the IC (Figures 7 and 8) was not cooled sufficiently to deactivate neuronal function and therefore one would not expect the structures beneath it, including the DNLL, to be deactivated either.

In previous studies where cooling was used to deactivate auditory cortex in guinea pig a reduction in cochlear temperature was observed of up to 4°C on the side ipsilateral to the cooled cortex attributed to the proximity to the bulla of the jugular vein into which blood from the cortex ultimately drains (Coomber et al., 2011). We found that cooling the IC lead to a minimal effect on the cochlea, with the temperature reduced by less than 2°C from the animal's core temperature, and a similar change in the cochlear nucleus. This difference is presumably explained by differences in the vascular system serving the IC and the cortex, particularly the relative blood flow and venous drainage.

Since the aim of our application is to test the effect of unilateral IC deactivation on neural response to sounds, the spread of cold to the contralateral IC is a major concern. We found that cooling the IC does produce a small fall in temperature on the contralateral side, but always less than 5°C even after a prolonged cooling cycle of >30 min. Such effects can also be mitigated by keeping the cooling cycles as short as possible and using the warmest effective cooling temperature. However, our measurements in Figures 2 and 3, made after aspirating the cortex, underestimate the temperature of the contralateral IC when the cortex is intact. Removal of the cortex led to a fall in temperature of about 2°C at a depth of 1 mm in the uncooled IC. Without the cortex, the mean temperature of the contralateral IC during cooling was close to 30°C, but with the cortex intact its insulating and warming effect should maintain the temperature of the IC at ~32°C. This is significant because when recording activity in the IC contralateral to the cooled IC our electrode penetrations are made through the cortex. This suggests the changes we observe in firing rate and temporal firing pattern are the consequence of blocking neural mechanisms in the contralateral, cooled IC, rather than direct cooling of the recorded neurons. Further evidence in support of this argument is our observation that changes in firing occur in the absence of significant changes in spike width, a direct measure of the effect of cooling (Volgushev et al., 2000b; Girardin and Martin, 2009).

Neural measurements of activity in the IC contralateral to the cooled IC provide evidence for an interaction between the two structures. These include changes in overall firing rate and in the temporal response properties of neurons that recover to pre-cool control values on rewarming. In addition, we found evidence for other changes in sound driven responses that will be reported separately. The summary data (Figure 11) show elevations and reductions in firing rate occur following deactivation of the contralateral IC. These are consistent with the findings reported when the contralateral IC is deactivated by drug injection (Malmierca et al., 2005). Such findings suggest that the commissural connections between the ICs mediate both excitatory and inhibitory effects, and the latter could be mediated by mono or disynaptic connections (Moore et al., 1998; Smith et al., 1998; Malmierca et al., 2005).

Given the extensive commissural fibers that interconnect the two ICs, blockade of these connections seems the most likely explanation for the changes we observed while cooling the other IC. However, an alternative mode of influence that could contribute to these effects is the modulation of descending activity from the IC since the neurons giving rise to descending fibers will also be deactivated by cooling. In guinea pig the IC sends descending projections to several upstream brainstem centers, both ipsilaterally and contralaterally (Malmierca et al., 1996; Schofield, 2001, 2010). Could the removal of descending input to these centers by cooling one IC explain the changes in neural activity we observed in the contralateral IC? Although we cannot rule out this possibility, evidence from LFP recordings suggests otherwise (Figure 12). In our recordings the first prominent peak in the LFP (particularly evident in responses to diotic stimuli or stimuli applied to the ear contralateral to the recorded colliculus) had a latency of about 5–6 ms, about 1 ms shorter than the earliest spike latencies recorded in guinea pig IC. This latency also matched previous latency measurements of the P5 wave in the auditory brainstem response of guinea pig, and to LFPs recorded intracollicularly (Dum et al., 1981; Harrison and Palmer, 1984). It seems reasonable, therefore, to suggest that the first peak in the LFP corresponds to the latency of the incoming afferent volley to the IC and the synaptic activity that it generates. This short latency peak was minimally changed by cooling the other IC, both in terms of its amplitude and its latency. In contrast, the later components in the LFP, likely reflecting the activity of intracollicular processing, showed marked changes in morphology and latency with cooling. These changes could therefore represent the modulation of a commissurally-mediated influence over processing within the IC.

It could be argued that the LFP is not sufficiently sensitive to reflect the effect of descending control mediated by the IC, so although the conservative position is to assume that the effects of cooling one IC on the other are mediated by more than one pathway, given the strength of commissural input it is likely to be the dominant component. This limitation applies not only to cooling deactivation but to any method in which the circuitry of the IC is globally deactivated. If, as evidence suggests, different populations of neurons in the IC give rise to the descending and commissural projections (Okoyama et al., 2006), it may eventually be possible to selectively deactivate the descending and commissural systems, for example, by using optogenetic methods (Yizhar et al., 2011).

A further route whereby deactivation of one IC could affect the other is via a cortico-thalamic loop. Tracer studies show that the commissure contains some fibers that project from the IC to the contralateral auditory thalamus (Aitkin and Phillips, 1984) and there are descending connections from the contralateral cortex to the IC that probably involve the commissure (Winer et al., 2002; Bajo and Moore, 2005; Bajo et al., 2007). Deactivation of the IC would partially remove the drive to these centers and therefore could reduce the influence of their descending input to the contralateral IC. The contribution of these components to the commissure is, however, relatively small. Furthermore these fibers terminate mainly in the dorsal cortex and are therefore less likely to exert a direct effect in the central nucleus.

We conclude that cooling is a viable means of deactivating the IC and of studying the interaction between the two ICs. The rapid onset and reversibility of cooling, combined with the absence of any need for interference with the preparation during the cooling process, offer major advantages over drug injection methods. Like all deactivation methods that do not permit selective deactivation of different neuronal types, there is currently no way of isolating the contribution of commissural input from effects of descending connections to lower brainstem structures. Our results point to modulation of commissural input being the major effect of cooling the contralateral IC. This method produces changes in the response properties of IC neurons consistent with those observed with inactivation by drug injection, but offers the potential for more in depth studies of the role of the CoIC in auditory processing.

### Conflict of interest statement

The authors declare that the research was conducted in the absence of any commercial or financial relationships that could be construed as a potential conflict of interest.

## Abbreviations

CF: characteristic frequency
CoIC: commissure of the inferior colliculus
DNLL: dorsal nucleus of the lateral lemniscus
EI: excitatory-inhibitory
FRA: frequency response area
IC: inferior colliculus
IQR: interquartile range
ISI: interspike interval
ISIH: interspike interval histogram
LFP: local field potential
PSTH: post stimulus time histogram
SPL: sound pressure level.
